# The Impact of workplace Harassment on Mental Health among healthcare workers

**DOI:** 10.1192/j.eurpsy.2025.1559

**Published:** 2025-08-26

**Authors:** A. Aloui, R. Ghammem, M. Bouhoula, I. Kacem, Y. Tebouri, A. Chouchane, A. Koubaa, M. Maoua, A. Brahem, O. El Maalel, J. Maatoug, S. Chatti, H. Kalboussi

**Affiliations:** 1 Occupational Medecine Department, University Hospital of Farhat Hached, sousse, Tunisia; 2 University of Sousse, Faculty of Medecine Ibn El Jazzar, 4000, Sousse, Tunisia; 3 Research laboratory LR 19SP03, Study of the risks and prospects for the prevention of non-communicable diseases in the workplace; 4 Epidemiology Department, University Hospital of Farhat Hached; 5 University of Sousse, Study of the risks and prospects for the prevention of non-communicable diseases in the workplace; 6 Occupational medecine Department, University Hospital La Rabta, sousse, Tunisia, Tunisia

## Abstract

**Introduction:**

Workplace harassment is one of the main psychosocial risks, leading to suffering at work or a lack of well-being. It can compromise one’s sense of safety and dignity and leave lasting scars on mental health.

**Objectives:**

Determine the prevalence of workplace harassment in healthcare environments and evaluate its impact on mental health.

**Methods:**

This is across-sectional study conducted among health professionals at an University Hospital in Tunisia. Data was collected using a pre-established synoptic form on the social and professional characteristics of the participants. Workplace harassment was assessed using the LIPT questionnaire.

**Results:**

Of the 500 healthcare workers included in this study period, 362 respended to the questionnaire representing a response rate of 72.4%. The average age of the participants was 37.3±8.71 years, with women predominating (59.4%). The majority of participants were paramedical staff (54.4%). Of the participants, 101 (27.9%) had at least one comorbidity. The most common medical conditions were hypertension (10.5%). As for psychiatric history, which was present in 21.8% of participants, depression was the most common pathology (53.1%). The prevalence of harassment was 19.9%. Both men and women were harassed in 47.9% of cases, 66.7% by their hierarchical superior, and 72.2% by their colleagues. Our results show that 27.4% of subjects reporting palpitations were harassed, compared with 14.8% in the group without palpitations (p=0.003). Similarly, the frequency of harassment was significantly higher in participants with a lump in the throat. Subjects with fatigue were also significantly more harassed than subjects without this complaint (25.5% vs. 12%, p=0.002). Anxiety and competition problems were 4.27 (CI95%(2.3-7.91), p<0.001) and 4.41 (CI95%(2.32-8.39), p<0.001) times more frequent in people suffering harassment.

**Conclusions:**

The prevalence of harassment among health professionals is not negligible, and the repercussions of this phenomenon are frequent. Given the extent of this emerging concept, preventive measures must be proposed and studied in order to put an end to its harmful effects.

**Disclosure of Interest:**

None Declared

